# UTMD inhibit EMT of breast cancer through the ROS/miR-200c/ZEB1 axis

**DOI:** 10.1038/s41598-020-63653-w

**Published:** 2020-04-20

**Authors:** Dandan Shi, Lu Guo, Xiao Sun, Mengmeng Shang, Dong Meng, Xiaoying Zhou, Xinxin Liu, Yading Zhao, Jie Li

**Affiliations:** Department of ultrasound, Qilu Hospital of Shandong University, Jinan, 250012 China

**Keywords:** Molecular biology, Oncology, Nanoscience and technology

## Abstract

As a potential drug/gene delivery system, the ultrasound-targeted microbubble destruction (UTMD) system can be used as a vehicle as well as increasing the permeability of biological barriers to enhance the effect of tumor treatment. However, the effect of UTMD in the tumor EMT process is unknown. In this study, we aimed to investigate the potential and mechanism of UTMD induced oxidative stress in inhibiting EMT of breast cancer. Human breast MDA231 cells were treated with microbubble (MB), ultrasound (US) and UTMD, respectively. The generation of oxidative stress, the levels of miR-200c, ZEB1 and vimentin, and the numbers of migratory cells were evaluated quantitatively and qualitatively by the measurement of intracellular reactive oxygen species (ROS), qRT-PCR, western blot assay, and transwell assay. Then, to evaluate the role of UTMD-induced oxidative stress and miR-200c in the epithelial-mesenchymal transition (EMT) inhibition, the ROS scavenger N-acetyl-L-cysteine (NAC) and miR-200c inhibitor were used before UTMD treatment. We found that UTMD induced oxidative stress, upregulated the expression of miR-200c, downregulated the expression of ZEB1 and vimentin and suppressed the MDA231 cell migration. The addition of NAC and miR-200c inhibitor had an opposite impact on the expression of miR-200c and ZEB1, thus hindered the effects of UTMD on MDA231 cells EMT. In conclusion, UTMD can inhibit the EMT characteristics of MDA231 cells. The mechanism may be related to the regulation of the miR-200c/ZEB1 axis through the generation of ROS induced by UTMD, which may provide a new strategy to prevent the tumor cells EMT under UTMD treatment.

## Introduction

The metastasis of cancer is a complicated process, and is the main cause of the patients’ death. Epithelial–mesenchymal transition (EMT) is a key regulator of aggressive invasion and metastasis in tumorigenesis^1,2^. During the EMT process, the epithelial cells lose the apical-basal polarity and cell-cell adhesion and get mesenchymal characteristics^3^. The major EMT markers comprise epithelial markers, such as E-cadherin, and mesenchymal markers, such as vimentin, N-cadherin, and fibronectin. Zinc finger E-box binding homeobox transcription factor 1 (ZEB1) is a critical EMT-related transcription factor (EMT-TF). ZEB1 knockdown represses vimentin expression, upregulated E-cadherin expression, and inhibited cell proliferation and metastasis^4^. Another central regulator of EMT is the microRNA-200 (miR-200) family, which consists of 5 members (miR-200a, -200b, -200c, -141 and -429)^5^. Mounting evidence suggests that ZEB1 and miR-200c reciprocally control their expression through a negative regulatory loop, and miR-200c repression or ZEB1 expression has been associated with a worse prognosis in several carcinomas, such as breast and ovarian cancer^5–7^.

Ultrasound (US) imaging is a real-time imaging technique which allows visualization of organ structure. Microbubbles (MBs) are used as ultrasonic contrast agents which increase the image contrast due to the acoustic mismatch between the gas and the surrounding vascular structures^8^. The ultrasound-targeted microbubble destruction (UTMD)-mediated drug/gene delivery system has shown a great potential on cancer therapy. In recent years, numerous studies *in vitro* and *in vivo* have demonstrated that UTMD-mediated drug/gene delivery improves tumoricidal effects, reduces toxicity of chemotherapeutics, reverses drug resistance of tumor cells, and assists other cancer therapies^9–11^. This has opened a new avenue for molecular diagnosis and therapy integration purposes. Exposure of tumor cells to ultrasonically activated MBs can not only increase biological barrier (cell membrane and endothelial layer) permeability through sonoporation, but also destroy the tumor microenvironment directly and mechanically through the UTMD-induced thermal effects and oxidative stress^12–14^. Oxidative stress occurs when the ultrasonic-mediated inertial cavitation produces free radicals and elevates intracellular reactive oxygen species (ROS) levels. It has been reported that the activation of ionic channels, formation of ROS, and influx of calcium ions induced by UTMD-mediated sonoporation, play important roles in the mechanisms of cell membrane permeabilization^14–17^.

ROS plays a causal role in a variety of pathologic conditions, including ischemia, ischemia/reperfusion (I/R) injury, diabetes, and aging^18,19^. ROS has been shown to affect cell signaling, triggering apoptosis, cell senescence and endothelial dysfunction^20^. Oxidative stress can modulate the expression level of miRNAs^21,22^. Fabrizio *et al*.^23^ revealed that ROS-induced miR-200c expression played a role in disrupting the SIRT1/FOXO1/eNOS regulatory loop, which involves functionally related proteins of endothelial function, cell senescence, and oxidative stress endurance. The MiR-200 family upregulated by oxidative stress was also reported by Magenta *et al*.^24^, who reported that miR-200c was strongly modulated by ROS in endothelial cells (EC), and that miR-200c over-expression affected EC proliferation, senescence, and apoptosis through downregulating its target protein ZEB1.

Although the efficiency of UTMD-mediated sonoporation in permeabilizing the biological barriers has been demonstrated, the exact mechanism behind the action of US and MBs has not been completely elucidated despite a number of proposed theories^13^. Furthermore, the effect of UTMD in inhibiting EMT has not been tested so far. In the present study, we studied the role of UTMD-induced oxidative stress on the tumor EMT process and investigated that the generation of ROS induced by UTMD inhibited EMT through the regulation of miR-200c/ZEB1 axis.

## Results

### The effect of UTMD treatment on EMT of MDA231 cells

**Figure 1:**
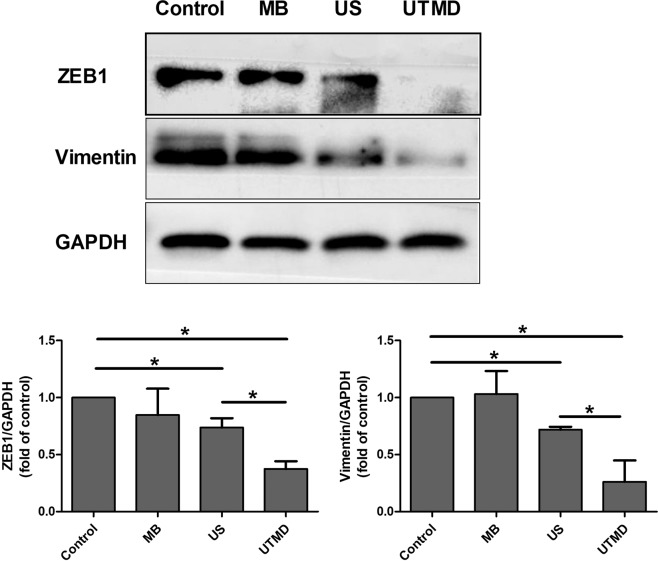
Western blot analyses of the ZEB1 and Vimentin protein expression in MDA231 cells of control and treated cells. Data are represented as mean ± SD (n = 3). The expressions of ZEB1 and vimentin were significantly lower in UTMD group than in the other groups (*P < 0.05).

To analyze whether UTMD regulated the migrative capability of tumor cells by inhibiting EMT, we detected the expressions of EMT-related protein in MDA231 cells of control, MB, US, and UTMD groups (Fig. 1). The original, unprocessed blots of the proteins from three times replicate experiments were showed in Figures S1–S9. The expressions of ZEB1 and vimentin were downregulated in the US and UTMD groups as compared to the control group (*P* < 0.05). Compared with the US group, The expressions of ZEB1 and vimentin of the UTMD group were significantly decreased (*P* < 0.05). Cells treated with MB alone did not exhibit any difference in the ZEB1 and vimentin expression to those of the control group.

**Figure 2:**
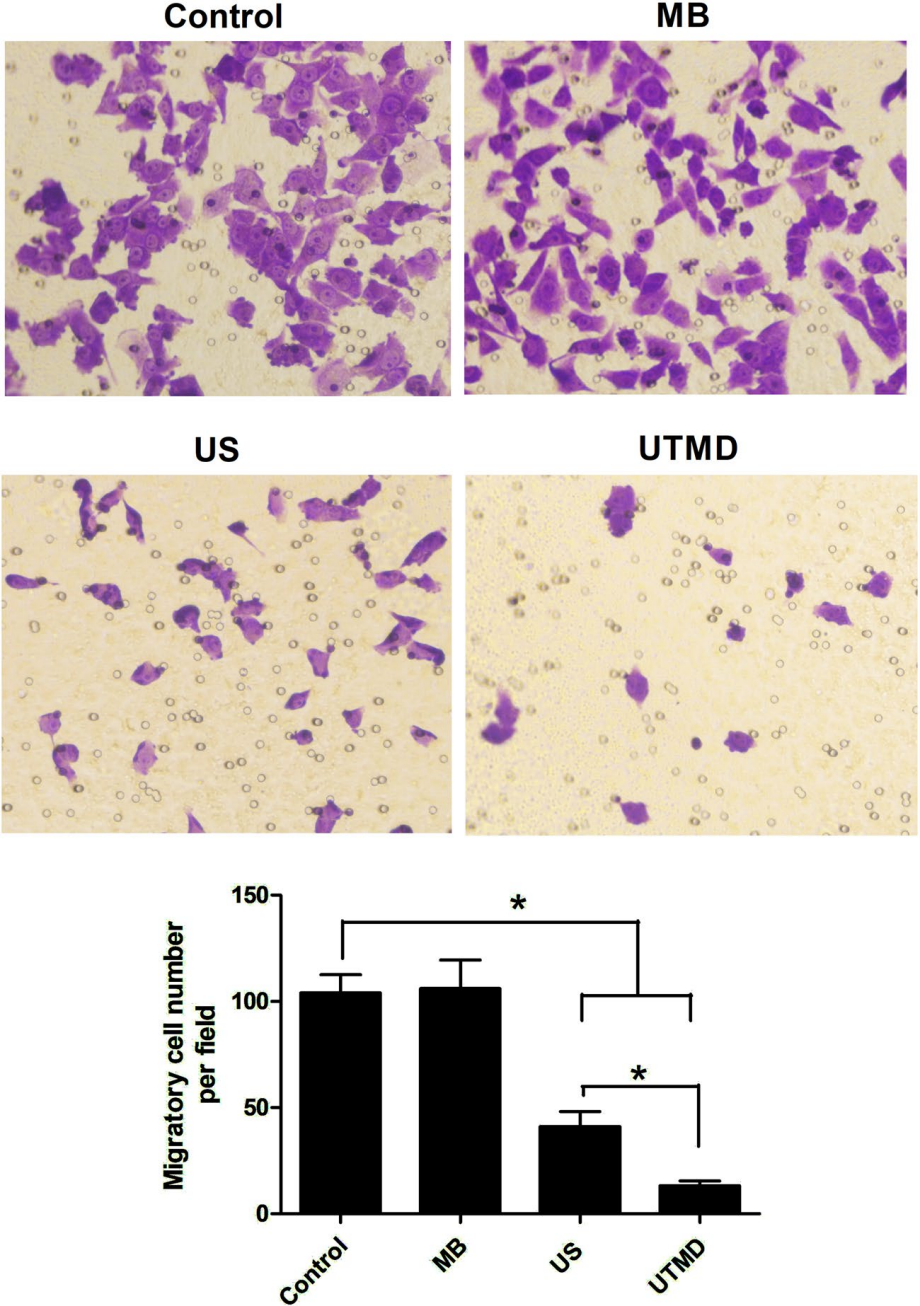
Analyses of changes in cell migration after different treatments on MDA231 cells using a transwell migrated assay. Data are represented as mean ± SD (n = 3). The number of migrated cells decreased significantly under US and UTMD treatment compared with the control group (*P < 0.05).

Meanwhile, the transwell assay results showed that a significant decrease of cell migration was observed in the US alone (*P* < 0.05 vs. control). The UTMD treatment further decreased the cell migration to a lower level (*P* < 0.05 vs. control), as shown in Fig. 2. Compared with the US group, the migratory cell number of the UTMD group was significantly decreased (*P* < 0.05), and no significant difference was found between the MB group and the control group.

### UTMD induced ROS generation

**Figure 3:**
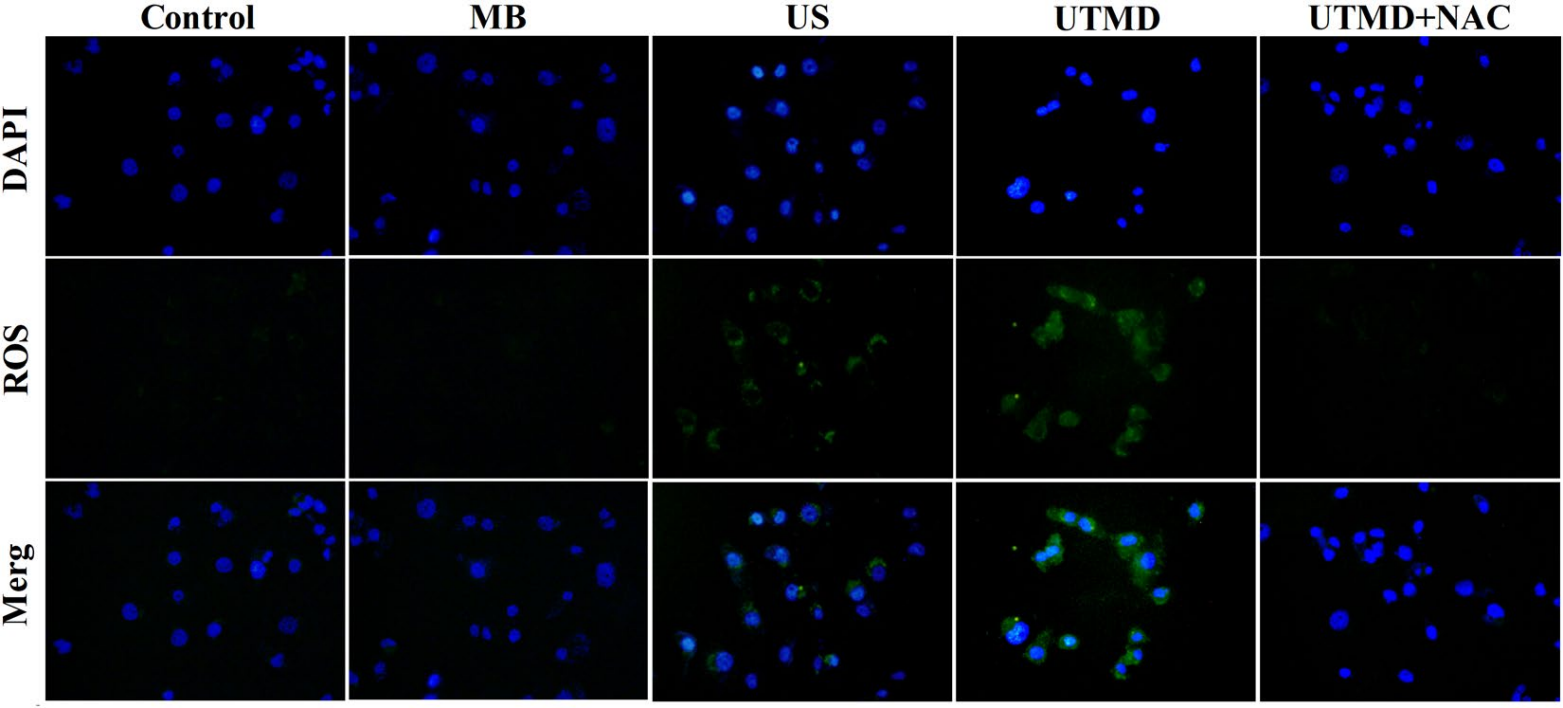
Representative fluorescent images of production of DCF due to ROS by the MDA231 cells after various conditions of stimulation. “Control” represents the group without treatment, “MB” represents the group with microbubble, “US” represents the group with ultrasound treatment, “UTMD” represents the group with UTMD treatment, and “UTMD + NAC” represents the group with NAC pre-incubation before the UTMD treatment.

**Figure 4:**
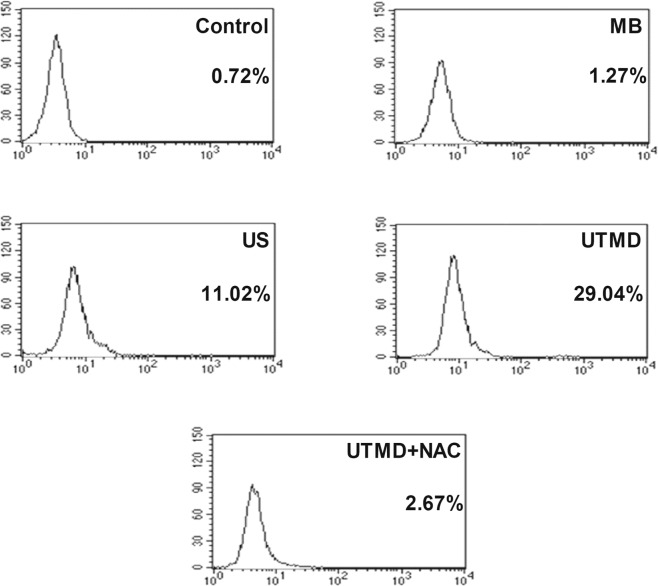
Flow cytometric analysis of levels of intracellular ROS after various conditions of stimulation by flow cytometry. The ultrasound treatment was set at an acoustic intensity of 1.0 W/cm^2^ for 30 sec. The MB concentration was set at 20%.

In this study, after the MDA231 cells were stimulated by various conditions, intracellular ROS generation of each group was observed by a fluorescent probe (DCFH-DA) assay using fluorescent microscopy and flow cytometry (FCM). Figure 3 showed representative fluorescence microscopic images of MDA231 cells exposed to MB, US, UTMD and UTMD + NAC. Compared to untreated controls, the ROS fluorescence of US-treated cells was slightly stronger, and that of UTMD treated cells was much stronger. The ROS fluorescence of the MB and UTMD + NAC group was close to that of the control group. As shown in Fig. 4, quantitative analysis for the intracellular ROS was performed using FCM, and the results were as follows: control: 0.72 ± 0.21%; MB: 1.27 ± 0.42%; US: 11.02 ± 0.75%; UTMD: 29.04 ± 0.67%; UTMD + NAC: 2.67 ± 0.22%. Results of US and UTMD groups demonstrated significant generation of intracellular ROS (*P* < 0.05 and *P* < 0.001). The results of MB and UTMD + NAC groups showed no significant difference compared to the control group (*P* > 0.05).

### UTMD modulates miR-200c expression

**Figure 5:**
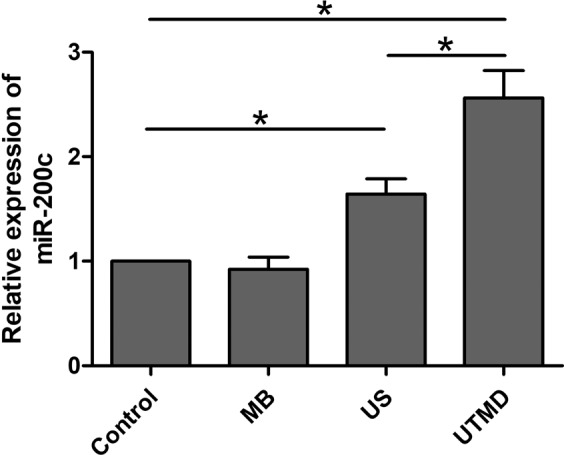
MiR-200c levels were determined in MDA231 cells after different treatments by qRT-PCR. Data are represented as mean ± SD (n = 3). The miR-200c expression of UTMD group was significantly upregulated than that in other groups (US vs control: 1.64 fold increased; UTMD vs control: 2.56 fold increased) (*P < 0.05).

Quantitative reverse transcription polymerase chain reaction (qRT-PCR) was used to examine the expression of miR-200c after various conditions of stimulation for 24 h. The results revealed that the MDA231 cells treated with US and UTMD showed significant upregulation of miR-200c compared to cells of the control group (US vs control: 1.64 fold increased, *P* < 0.05; UTMD vs control: 2.56 fold increase, *P* < 0.05), and the cells treated with MB alone did not show any significant changes in miR-200c expression. In UTMD groups, the expression of miR-200c was significantly lower than that in US group (Fig. 5). These observations demonstrated that the US treatment slightly modulated miR-200c expression, while the UTMD treatment had a significant impact on the miR-200c expression.

### UTMD modulates the EMT process through the regulation of the ROS/miR-200c/ZEB1 axis

**Figure 6:**
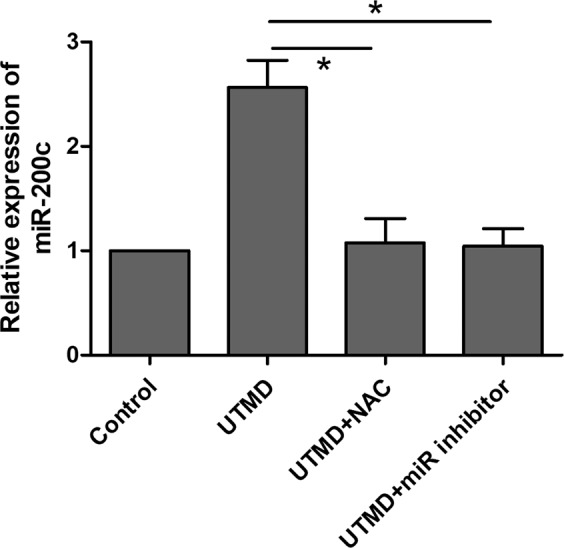
The ROS scavenger NAC and miR-200c inhibitor were used before UTMD treatment. MiR-200c expression in MDA231 after treatment with UTMD combined with NAC or miR-200c inhibitor was significantly decreased compared with the UTMD group. Data are represented as mean ± SD (n = 3). (*P < 0.05).

**Figure 7:**
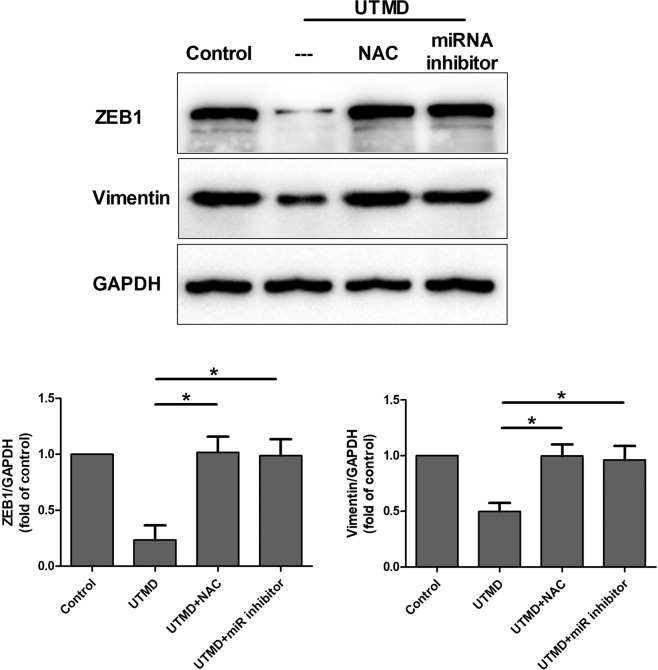
The EMT markers ZEB1 and vimentin in MDA231 cells after treatment with UTMD combined with NAC or miR-200c inhibitor analyzed by western blot analysis and statistically analyzed compared with the UTMD group. Data are represented as mean ± SD (n = 3) (*P < 0.05).

**Figure 8:**
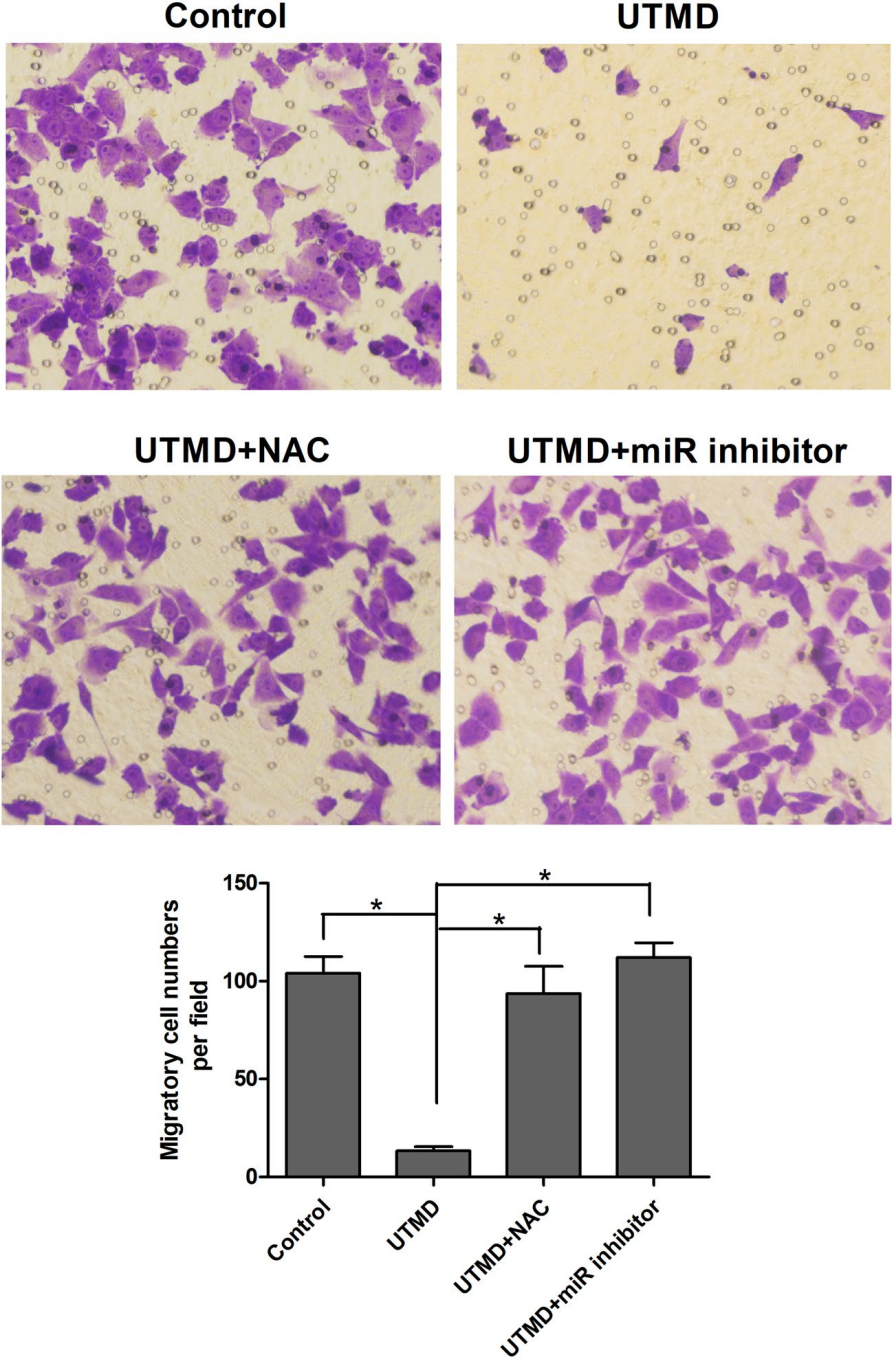
MDA231 cells of the UTMD + NAC and UTMD + miR inhibitor groups exhibited EMT characteristics. Compared with the UTMD group, the migrator cell numbers of UTMD + NAC and UTMD + miR inhibitor groups increased significantly, which was similar with the control group (*P < 0.05).

In order to identify the role of the miR-200c/ZEB1 axis in tumor EMT inhibition under UTMD treatment and their regulation by UTMD-induced oxidative stress, miR-200c and ZEB1 expression has been investigated by qRT-PCR and western blot assay in the control, UTMD, UTMD + NAC and UTMD + miR inhibitor groups. The qRT-PCR showed no significant change in miR-200c level among the control, UTMD + NAC, and UTMD + miR inhibitor groups. The level of miR-200c under UTMD treatment showed a significant increase compared with the control, UTMD + NAC and UTMD + miR inhibitor groups (all *P* < 0.05) (Fig. 6). The western blot assay showed that ZEB1 and vimentin expressions of control, UTMD + NAC, and UTMD + miR inhibitor groups showed no significant difference between groups, while those of the UTMD group were significantly decreased compared with the control, UTMD + NAC, and UTMD + miR inhibitor groups separately (Fig. 7). The original, unprocessed blots of the proteins from three times replicate experiments were showed in Figures S10–S18. As shown in Fig. 8, the transwell assay results revealed that a significant decrease of cell migration was observed in the UTMD group (*P* < 0.05 vs. control, UTMD + NAC, UTMD + miR inhibitor), and no significant difference was found among the control, UTMD + NAC, and UTMD + miR inhibitor groups (all *P* > 0.05).

## Discussion

Various studies have proved that UTMD suppresses the progression of carcinoma, and sonoporation-induced biological barrier permeability has emerged as a pivotal mechanism of UTMD in tumor progression repression^9,14,25^. To explore whether there is any other mechanism of UTMD-inhibited tumor progression, we investigated the role of UTMD in inhibiting breast cancer EMT and tried to reveal its mechanism. Firstly, we demonstrated the tumor EMT inhibition effect of UTMD in the MDA231 cells. Western blot assay demonstrated that UTMD can almost completely inhibit the expression of ZEB1 and vimentin. The transwell assay supported the conclusion from the point of the tumor cell migratory ability. It proved that UTMD can suppress migration of MDA231 cells, and combination treatment with ultrasound and microbubbles is more efficient than ultrasound alone, which indicates that the UTMD can potentially be used in EMT inhibition.

Secondly, it is necessary to explore the possible underlying mechanisms on inhibition of tumor cell EMT by the UTMD treatment. Oxidative stress as one of the main mechanisms for UTMD-induced bio-effects has been proposed^13,14,26^. The increase of intracellular ROS represents the increase of oxidative stress. Herein, we investigated the effect of UTMD on oxidative stress through detecting intracellular ROS levels in the MDA231 cells under various treatments. Results in Figs. 3 and 4 demonstrated that the ultrasound influenced the generation of intracellular ROS, and UTMD had a more significant impact on that. As an antioxidant, NAC has been used to scavenge the intracellular ROS. NAC pre-incubation before UTMD treatment strongly decreases the level of intracellular ROS, and only MB treatment has no influence on that. The above results confirm that UTMD treatment can induce oxidative stress and increase the level of intracellular ROS.

The expression level of miRNAs can be modulated by parameters of the tumor environment, such as hypoxia, nutrient deprivation, or oxidative stress^20^. MiR-200c, as a functionally related miRNA of EMT, has been proved to be upregulated by oxidative stress^23,24^. Based on the above results of UTMD inducing oxidative stress, we tested the expression of miR-200c under US and UTMD treatment. The results revealed that US and UTMD both showed the potential in upregulation of miR-200c. The levels of miR-200c in MDA231 cells of UTMD group were higher than those of control and US groups, which was similar to the findings in previous studies on the relationship of oxidative stress and miR-200c^27,28^. MiR-200c has been identified to play a central role in cancer aggressiveness by controlling tumor spreading and drug resistance through a double negative-feedback loop with the transcription factor ZEB1^5–7,29^. In the first part of the current study, we have verified that UTMD could significantly downregulate the ZEB1 expression. Therefore, we hypothesized that UTMD may inhibit EMT through the miR-200c/ZEB1 axis.

To address the role of oxidative stress and the miR-200c/ZEB1 axis in UTMD-induced tumor EMT inhibition, NAC and miR-200c inhibitor were added before the UTMD treatment to scavenge ROS and downregulate miR-200c expression, respectively. Compared with the UTMD group, the expression of miR-200c showed a significant decrease, while ZEB1 showed a significant increase in the UTMD + NAC and UTMD + miR inhibitor groups. When the ROS induced by UTMD was scavenged by NAC, the miRNA-200c expression significantly decreased, indicating that the UTMD-induced oxidative stress can increase the expression of miR-200c. Figure 7 showed that the ZEB1 expression significantly increased when the ROS induced by UTMD was scavenged by NAC, and the miR-200c induced by UTMD was inhibited by miR-200c inhibitor. These data suggested that UTMD-induced oxidative stress directly regulated the miR-200c/ZEB1 axis to inhibit the EMT in the MDA231 cells, which could be the potential molecular mechanisms underlying UTMD-inhibited EMT.

## Conclusions

In this study, we began by investigating the effect of UTMD in inhibiting the MDA231 cell EMT. Then, we focused on the relevant molecular mechanisms of UTMD-inhibited EMT. On one hand, UTMD was proved to induce the oxidative stress, upregulate the expression of miR-200c, downregulate the expression of ZEB1 and vimentin, and suppress the MDA231 cell migration. These findings indicated that UTMD inhibited the EMT characteristics of MDA231 cells. On the other hand, we studied the mechanisms of UTMD-induced oxidative stress on the tumor EMT process and demonstrated that ROS induced by UTMD inhibited EMT through the regulation of the miR-200c/ZEB1 axis. This new mechanistic insight could contribute to new strategies to prevent the tumor cell EMT under UTMD treatment and support the use of UTMD in cancer treatment in the future. However, the mechanism of UTMD treatment in suppressing the progression of carcinoma is complicated and not yet completely understood. More in depth studies *in vitro* and *in vivo* are certainly needed to verify this new mechanism.

## Materials and Methods

### Chemicals

Sonovue (Bracco Research SA, Geneva, Switzerland) is a suspension of stabilized sulfur hexafluoride microbubbles with phospholipid as the film material which is a commonly used ultrasound contrast agent in clinics. The Sonovue microbubbles were prepared by diluting the powder in a sterile 0.9% NaCl solution. An ROS assay kit was provided by Beyotime (Wuhan, China). Crystal violet was purchased from Sigma (Saint Louis, USA). The work solution of NAC (Beyotime, China) was 100 mM diluted by deionized water.

### Cell culture

The human breast tumor cell line MDA-MB-231 (MDA231) was obtained from the Chinese Academy of Sciences Cell Bank (Shanghai, China). Cells were cultured in DMEM medium (HyClone, Logan, UT, USA) supplemented with 10% heat-inactivated fetal bovine serum (FBS) (Gibco, Carlsbad, USA), 100 IU/mL of penicillin and 100 mg/mL of streptomycin sulfate. All cells were cultured at 37 °C in 5% CO_2_. The cells used in the experiments were in log-phase.

### ***In vitro*ultrasound and microbubble treatment**

The MDA231 cells with a density of 4 × 10^5^ cells/well were seeded in 6-well plates for 24 h. Then, all samples were randomly divided into different groups: the control group (Control), microbubble group (MB), ultrasound group (US), ultrasound targeted microbubble destruction group (UTMD), UTMD with NAC group (UTMD + NAC) and UTMD with miRNA-200c inhibitor group (UTMD + miR inhibitor). For all groups except the control and MB, the ultrasound treatment was set at an acoustic intensity of 1.0 W/cm^2^ for 30 sec. The MB concentration of all groups except the control and US groups was set at 20%, according to our previously published study^30^. The cells of the UTMD + NAC group were preincubating in 1.8 mL serum-free DMEM medium containing 200 μL NAC solution for 1 h before treatment with UTMD.

### Measurement of intracellular ROS

The quantitative assessment of intracellular ROS was carried out employing oxidation sensitive fluorescent probe 2′,7′-dichlorodihydrofluorescein diacetate (DCFH-DA). The DCFH-DA diffuses into cells and was deactivated by esterase to form fluorescent 2′,7′-dichlorofluorescein (DCF)^31,32^. The MDA231 cells with a density of 4 × 10^5^ cells/well were seeded in 6-well plates for 24 h and treated with ultrasound and/or microbubbles. The cells without US and MB served as a negative control. The MDA231 cells in the culture wells were incubated with 10-μM DCFH-DA solutions at 37 °C for 0.5 h. Then, the cells were washed 3 times with phosphate-buffered saline (PBS). The DCF fluorescence imaging was acquired with a fluorescence microscope (Olympus DP72, Japan). The intracellular fluorescence intensity of DCF was measured by flow cytometry (FCM) at an excitation wavelength of 488 nm and an emission wavelength of 525 nm.

### Cell transfection

MDA231 cells of UTMD from the miRNA-200c-3p inhibitor group were plated in 6-well plates (4 × 10^5^ cells/well) overnight. Then, the cells were transfected with 50 nM of miRNA-200c-3p inhibitor (Ribobio, Guangzhou, China) at the concentration of 50 nM using EndoFectin-MAX transfection reagent (GeneCopoeia, Rockville, USA) and Opti-MEM I reduced serum medium (Gibco, Carlsbad, USA) according to the manufacturer’s instructions. Cells were transfected with negative controls siRNA (NC) at the concentration of 5 nM as a control. At 4 h post-transfection, the MDA231 cells of the UTMD + miR inhibitor group were treated with UTMD.

### qRT-PCR analysis for miRNA-200c expression

Total RNA was extracted from cells using RNAfast200 kit (Fastagen, Shanghai, China Invitrogen) according to the manufacturer’s instructions and stored at −80 °C. After 4 μg of total RNA was reverse transcribed into cDNA, qRT-PCR was performed using an All-in-One miRNA qRT-PCR detection Kit (GeneCopoeia, Rockville, USA). The primers were synthesized by GeneCopoeia, Inc., and the results are shown in Table 1.

**Table 1:** Primers used for real-time PCR.

miRNA Genes	Catalog# (GeneCopoeiaTM)
has-miRNA-200c-3p	HmiRQP0301
hanRNA U6-2	HmiRQP9001

### Western blot analysis

MDA231 cells were collected and homogenized in lysis buffer (RIPA: PMSF = 100:1) for 20 min. The lysates were centrifuged at 12,000 rpm for 10 min. The total protein content of lysates was quantified using an Enhanced BCA Protein Assay Kit (Beyotime, China), with BSA as the standard. Protein lysates were denatured at 96 °C for 5 min after mixing with 20% sodium dodecyl sulfate (SDS-loading) buffer. The proteins (30–70 μg/lane) were separated by 10% SDS polyacrylamide gel electrophoresis (SDS-PAGE) gels, and then transferred onto nitrocellulose membranes (Merck Millipore, USA). In order to save the antibodies and experimental fund, the membranes were cropped to a suitable width according to the molecular weight of the proteins. This method is adopted in our laboratory. And then the membranes were blocked with western blocking buffer (Beyotime, China) for 60 min and incubated overnight at 4 °C with primary antibodies against vimentin (dilution 1:500, Millipore), ZEB1 (dilution 1:1,000, Affinity) and GAPDH (dilution 1:10,000, HuaBio). After washing with tris buffered saline (TBST), the membranes were incubated with either secondary horseradish peroxidase-conjugated anti-rabbit or anti-mouse immunoglobulin G (IgG) antibodies (Beyotime, China) followed by 3 additional washing steps with TBST. Bands were visualized on a luminescent image analyzer (Amersham Imager 600, GE; FluorChem E, ProteinSample) using chemiluminescence HRP substrate (Millipore, USA). Finally, the bands were quantified using Image-J software (NIH).

### Transwell assay

A transwell chamber (Corning Costar, USA) was used to perform migrative assay. Cells of 2 × 10^4^ were seeded into the upper chambers with serum-free medium, and 600 μL of the DMEM containing 10% FBS was added to the lower chambers and cultured for 18 h. The cells that migrated to the underside of the upper chambers were fixed with methanol for 15 min, stained for 20 min with 0.1% crystal violet, and washed with PBS. An optical microscope (100×) was used to observe and quantify the migrated cells, and 5 fields of view for each group were randomly selected and averaged.

### Statistical analysis

The experiments were done at least three times with biological replicates, and measurement data were expressed as mean values ± standard deviation. Differences between groups were assessed by Student’s 2-tailed t-test for independent samples. All data in the experiment were analyzed using SPSS 21.0 software. *P* < 0.05 was considered statistically significant.

## Supplementary information

Supplementary information.
