# Supplementary material for: UTMD inhibit EMT of breast cancer through the ROS/miR-200c/ZEB1 axis
Source: Sci Rep. 2020 Apr 20;10:6657. doi: 10.1038/s41598-020-63653-w (PMC7170845; doi:10.1038/s41598-020-63653-w)
Supplement: Supplementary file 1 — Supplementary information. [file 41598_2020_63653_MOESM1_ESM.pdf]

Supplementary Information

UTMD inhibit EMT of breast cancer through the ROS/miR-200c/ZEB1 axis

Dandan Shi[1], Lu Guo[1], Xiao Sun[1], Mengmeng Shang[1], Dong Meng[1], Xiaoying Zhou[1], Xinxin Liu[1], Yading Zhao[1], Jie Li[1*]

[1]Department of ultrasound, Qilu Hospital of Shandong University, Jinan 250012, China.

* Corresponding author: Jie Li, Department of Ultrasound, Qilu Hospital of Shandong University, Jinan 250012, China, Tel: 86-531-2166101, Fax: 86-531-86927544, E-mail: jieli301@163.com

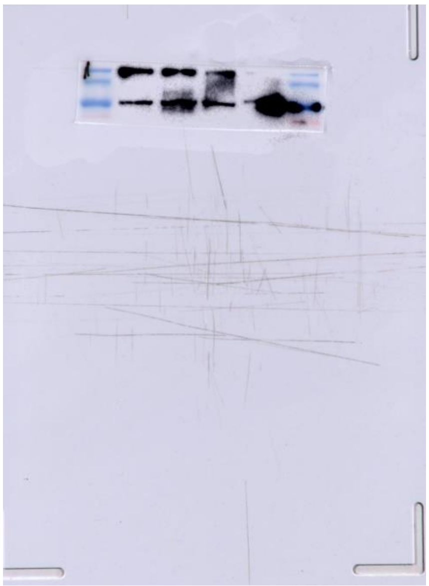

Fig. S1. The original, unprocessed blots of ZEB1 protein of Test 1 in Fig. 1. The upper row of the bands shows the expression of ZEB1. The lower row is nonspecific band. Band #1 indicates control group. Band #2 indicates MB group. Band #3 indicates US group. Band #4 indicates UTMD group. The blots of Fig. S1., Fig. S2. and Fig. S3. cropped from different gels of the same experiment.

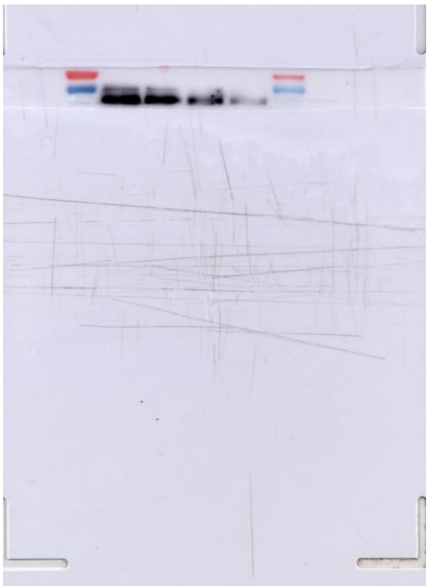

Fig. S2. The original, unprocessed blots of vimentin protein of Test 1in Fig. 1. Band #1 indicates control group. Band #2 indicates MB group. Band #3 indicates US group. Band #4 indicates UTMD group.

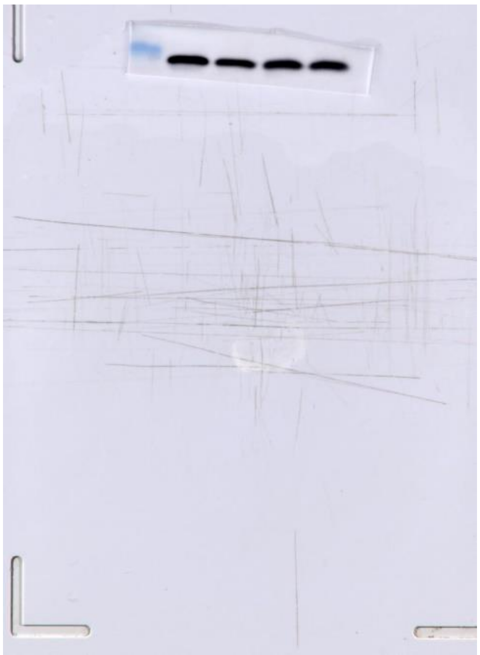

Fig. S3. The original, unprocessed blots of GAPDH protein of Test 1 in Fig. 1. GAPDH was used as an internal reference. Band #1 indicates control group. Band #2 indicates MB group. Band #3 indicates US group. Band #4 indicates UTMD group.

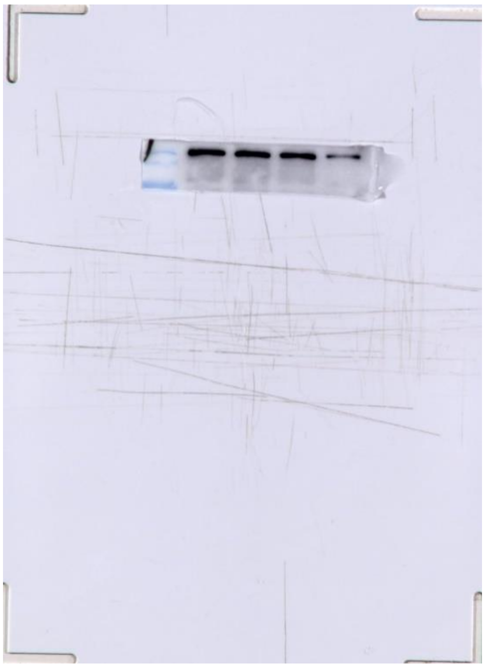

Fig. S4. The original, unprocessed blots of ZEB1 protein of Test 2 in Fig. 1. The upper row of the bands shows the expression of ZEB1. Band #1 indicates control group. Band #2 indicates MB group. Band #3 indicates US group. Band #4 indicates UTMD group.

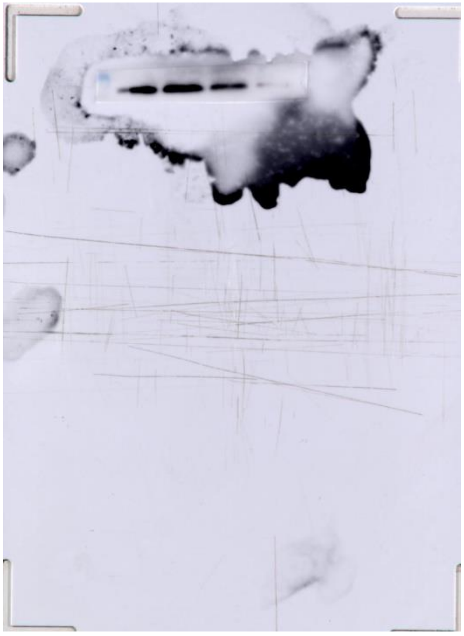

Fig. S5. The original, unprocessed blots of vimentin protein of Test 2 in Fig. 1. Band #1 indicates control group. Band #2 indicates MB group. Band #3 indicates US group. Band #4 indicates UTMD group.

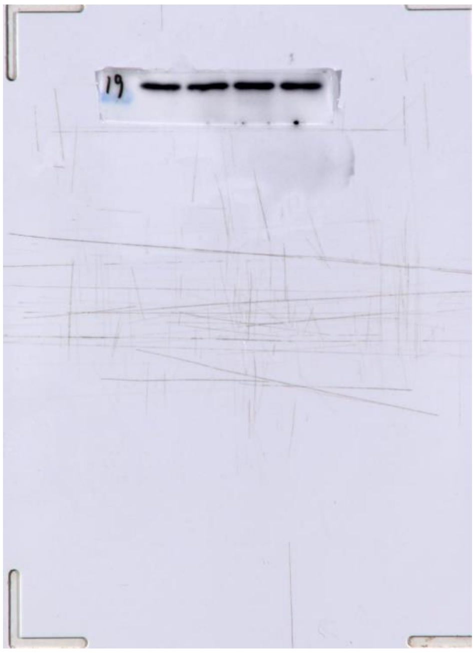

Fig. S6. The original, unprocessed blots of GAPDH protein of Test 2 in Fig. 1. GAPDH was used as an internal reference. Band #1 indicates control group. Band #2 indicates MB group. Band #3 indicates US group. Band #4 indicates UTMD group.

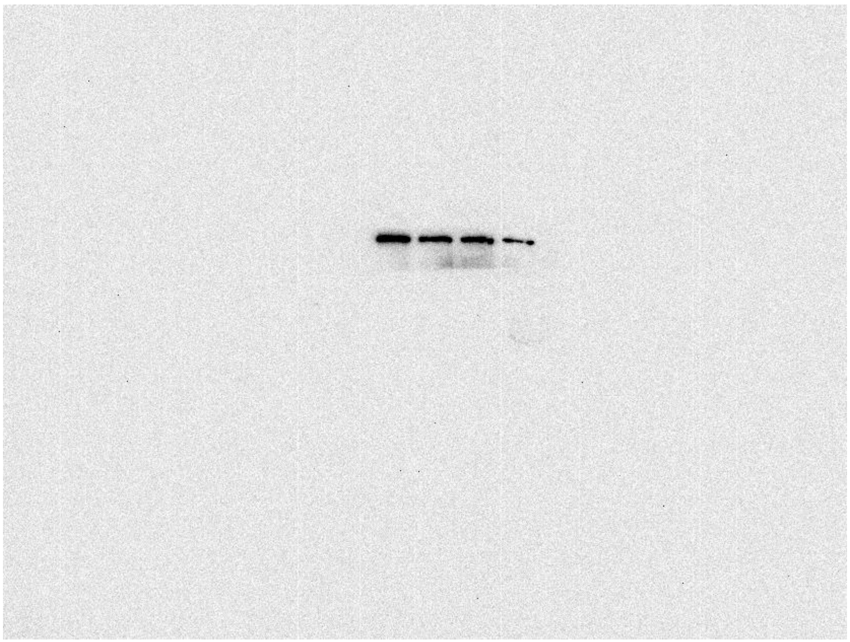

Fig. S7. The original, unprocessed blots of ZEB1 protein of Test 3 in Fig. 1. The upper row of the bands shows the expression of ZEB1. Band #1 indicates control group. Band #2 indicates MB group. Band #3 indicates US group. Band #4 indicates UTMD group.

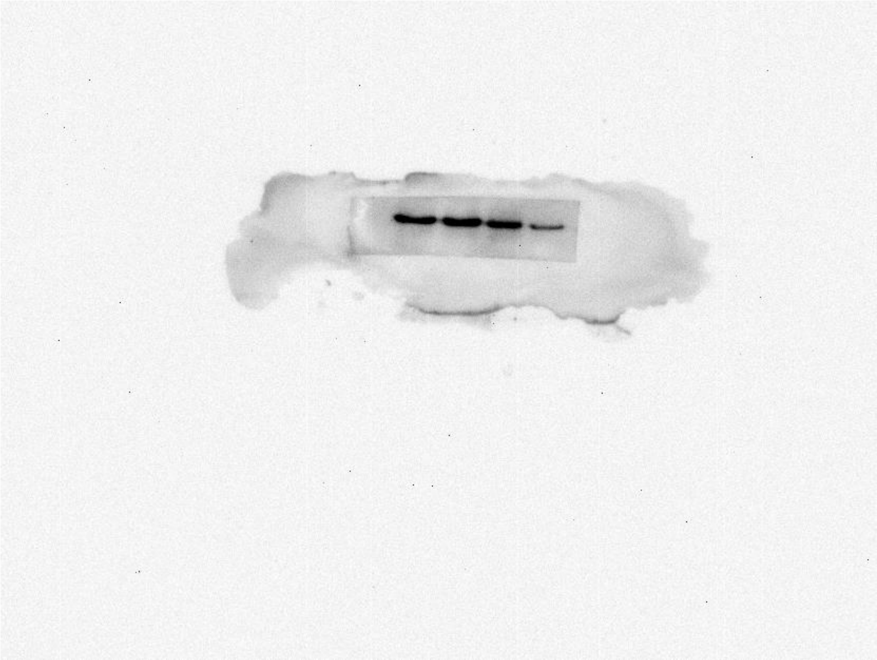

Fig. S8. The original, unprocessed blots of vimentin protein of Test 3 in Fig. 1. Band #1 indicates control group. Band #2 indicates MB group. Band #3 indicates US group. Band #4 indicates UTMD group.

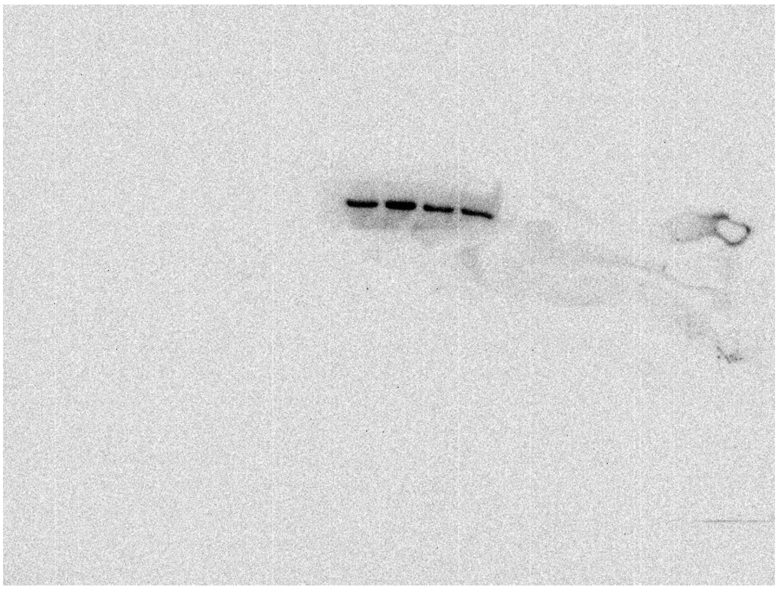

Fig. S9. The original, unprocessed blots of GAPDH protein of Test 3 in Fig. 1. GAPDH was used as an internal reference. Band #1 indicates control group. Band #2 indicates MB group. Band #3 indicates US group. Band #4 indicates UTMD group.

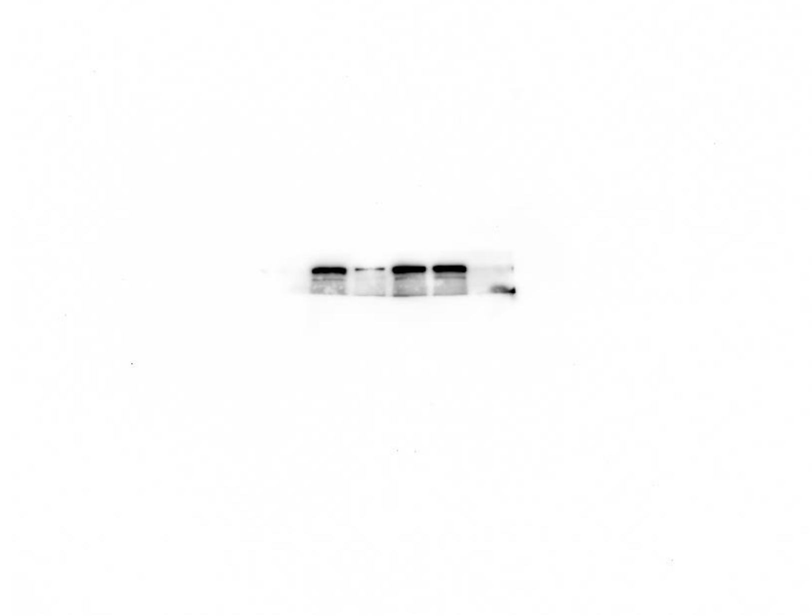

Fig. S10. The original, unprocessed blots of ZEB1 protein of Test 1 in Fig. 7. Band #1 indicates control group. Band #2 indicates UTMD group. Band #3 indicates UTMD+NAC group. Band #4 indicates UTMD+miR inhibitor group. The blots of Fig. S4., Fig. S5. and Fig. S6. cropped from different gels of the same experiment.

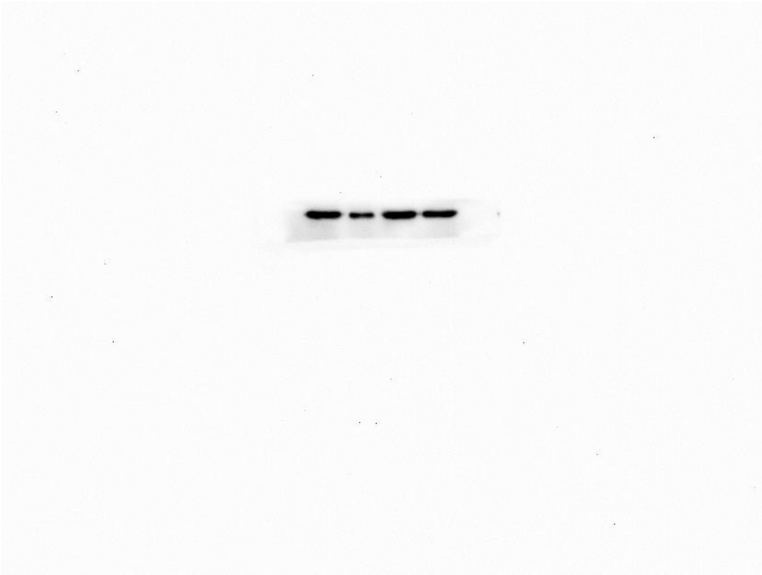

Fig. S11. The original, unprocessed blots of vimentin protein of Test 1 in Fig. 7. Band #1 indicates control group. Band #2 indicates UTMD group. Band #3 indicates UTMD+NAC group. Band #4 indicates UTMD+miR inhibitor group.

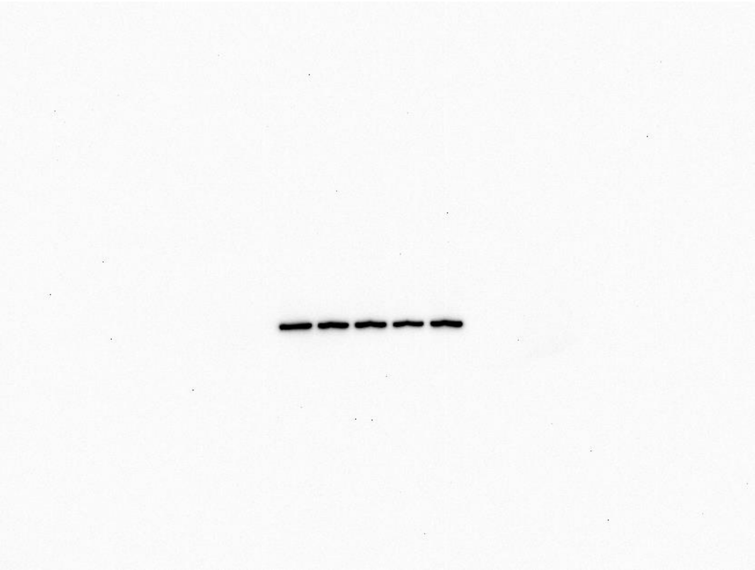

Fig. S12. The original, unprocessed blots of GAPDH protein of Test 1 in Fig. 7. GAPDH was used as an internal reference. Band #1 and Band #2 indicate repeated bands of control group. Band #3 indicates UTMD group. Band #4 indicates UTMD+NAC group. Band #5 indicates UTMD+miR inhibitor group. We showed the last four bands in the Fig. 7.

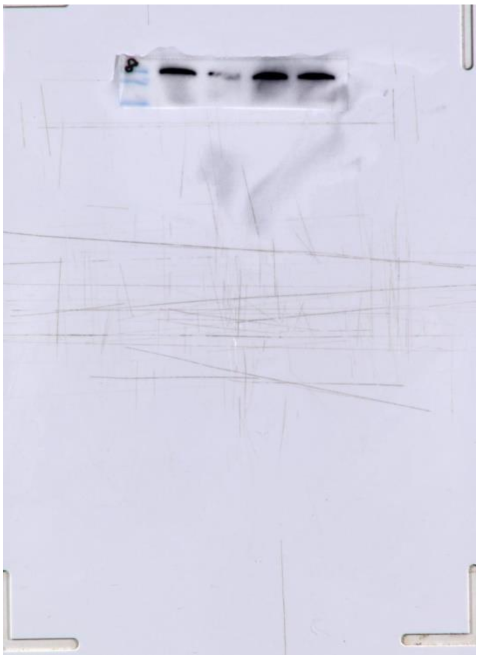

Fig. S13. The original, unprocessed blots of ZEB1 protein of Test 2 in Fig. 7. Band #1 indicates control group. Band #2 indicates UTMD group. Band #3 indicates UTMD+NAC group. Band #4 indicates UTMD+miR inhibitor group.

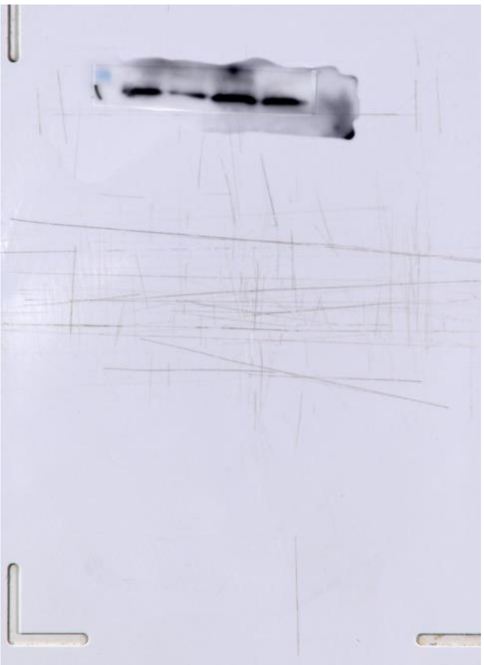

Fig. S14. The original, unprocessed blots of vimentin protein of Test 2 in Fig. 7. Band #1 indicates control group. Band #2 indicates UTMD group. Band #3 indicates UTMD+NAC group. Band #4 indicates UTMD+miR inhibitor group.

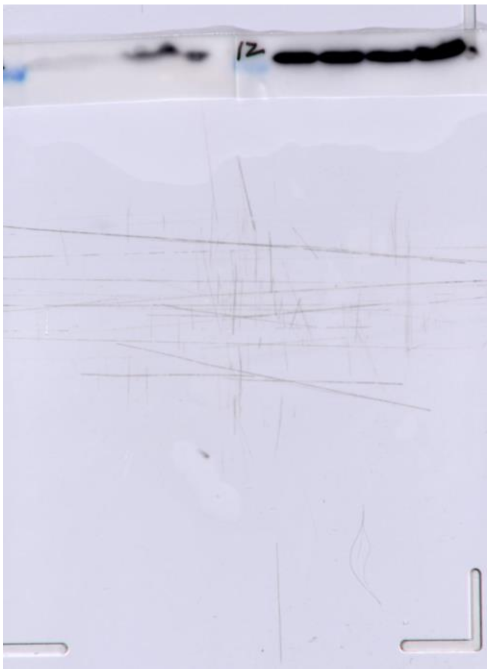

Fig. S15. The band on the right side is the original, unprocessed blots of GAPDH protein of Test 2 in Fig. 7. GAPDH was used as an internal reference. Band #1 indicates repeated bands of control group. Band #2 indicates UTMD group. Band #3 indicates UTMD+NAC group. Band #4 indicates UTMD+miR inhibitor group.

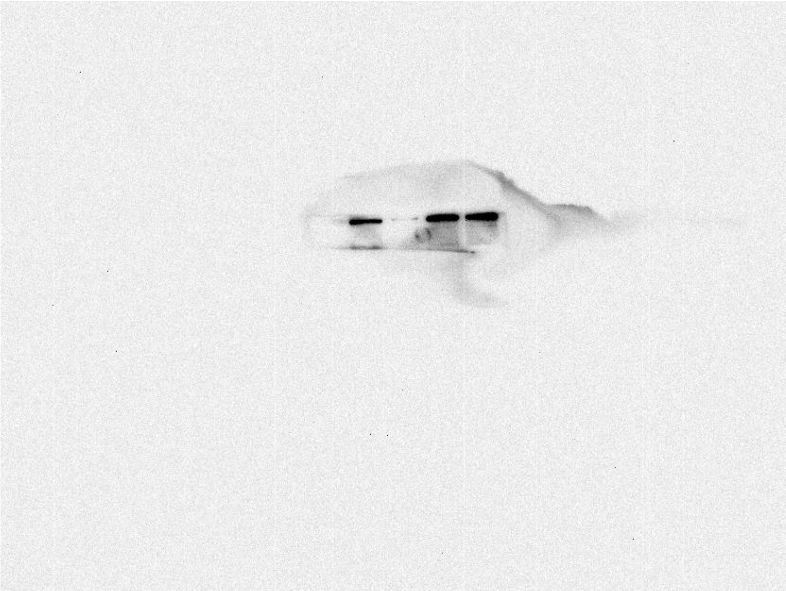

Fig. S16. The original, unprocessed blots of ZEB1 protein of Test 3 in Fig. 7. Band #1 indicates control group. Band #2 indicates UTMD group. Band #3 indicates UTMD+NAC group. Band #4 indicates UTMD+miR inhibitor group.

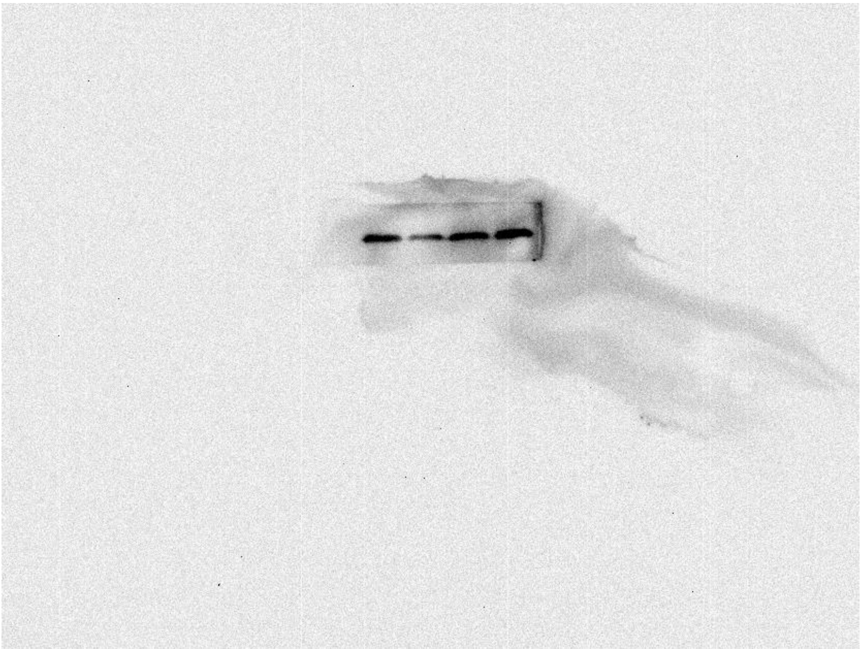

Fig. S17. The original, unprocessed blots of vimentin protein of Test 3 in Fig. 7. Band #1 indicates control group. Band #2 indicates UTMD group. Band #3 indicates UTMD+NAC group. Band #4 indicates UTMD+miR inhibitor group.

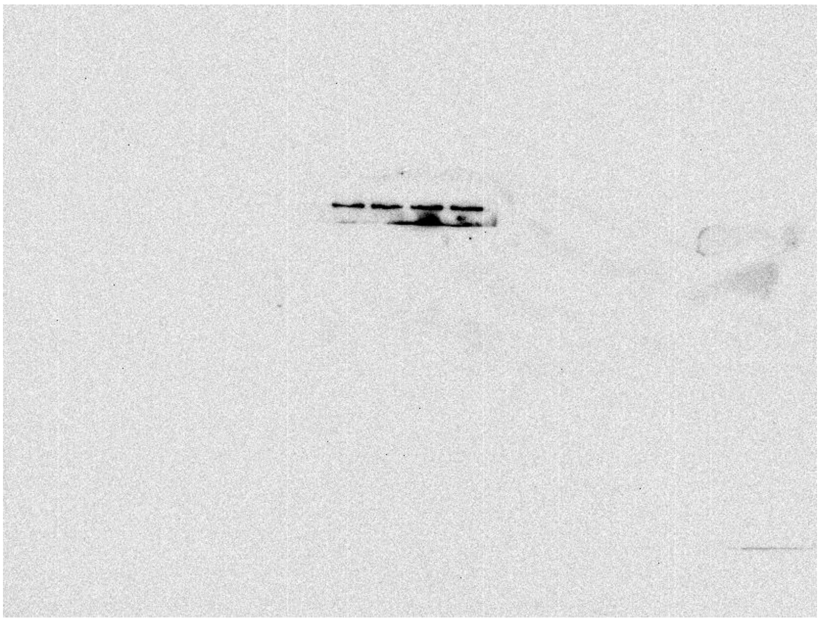

Fig. S18. The original, unprocessed blots of GAPDH protein of Test 3 in Fig. 7. GAPDH was used as an internal reference. Band #1 indicates repeated bands of control group. Band #2 indicates UTMD group. Band #3 indicates UTMD+NAC group. Band #4 indicates UTMD+miR inhibitor group.
